# More Letters about the Package Library

**Published:** 1915-03

**Authors:** 


### More Letters About the Package Library.

DECATUR, TEXAS, February 6, 1915.

*Mrs. F. E. Daniel, Austin, Texas.*

MY DEAR MRS. DANIEL: I believe your plan for a package library system is a sensible idea—a movement in the right direction—and it should mean much to the busy doctor. You are to be congratulated on your spirit and energy in this undertaking.

L. H. REEVES.

---

DAYTON, TEXAS, January 12, 1915.

*Mrs. F. E. Daniel, Austin, Texas.*

DEAR MRS. DANIEL: I think the "package library system" which you mention a great work and will be of inestimable value to the profession, especially for the doctors of the small towns. I heartily endorse the plan and think you deserve great credit for the undertaking.

Respectfully,

J. T. TADLOCK.

